# Early Structural Degradation of Dermal Elastic Fibers in Women with Mild Obesity Without Parallel Transcriptional Changes

**DOI:** 10.3390/jcm14155220

**Published:** 2025-07-23

**Authors:** Hiroko Makihara, Kazusa Kaiga, Toshihiko Satake, Mayu Muto, Yui Tsunoda, Hideaki Mitsui, Kenichi Ohashi, Tomoko Akase

**Affiliations:** 1 Department of Biological Science and Nursing, Graduate School of Medicine, Yokohama City University, Yokohama 236-0004, Japan; makihara.hiroko@twmu.ac.jp (H.M.); t186613a@yokohama-cu.ac.jp (K.K.); himitsu@yokohama-cu.ac.jp (H.M.); 2 Department of Biochemistry, School of Medicine, Tokyo Women’s Medical University, Shinjuku-ku, Tokyo 162-8666, Japan; 3 Department of Plastic, Reconstructive and Aesthetic Surgery, Faculty of Medicine, University of Toyama, Toyama 930-8555, Japan; toshi@med.u-toyama.ac.jp; 4 Lala Breast Reconstruction Clinic Yokohama, Yokohama 231-0015, Japan; m.mayu.u530@gmail.com (M.M.); ytsunoda0128@gmail.com (Y.T.); 5 Department of Human Pathology, Graduate School of Medical and Dental Sciences, Institute of Science Tokyo, Bunkyo-ku, Tokyo 113-8510, Japan; kohashi.pth1@tmd.ac.jp

**Keywords:** elastin degradation, body mass index, mild obesity, skin

## Abstract

**Background/Objectives:** Obesity is associated with various skin complications, yet its impact on dermal elastic fibers—key components maintaining skin elasticity—remains unclear, particularly in cases of mild obesity prevalent in East Asian populations. The aim of this study was to investigate whether mild obesity is associated with the early structural deterioration of dermal elastic fibers and alterations in elastin-related gene expression in Japanese individuals. **Methods:** Abdominal skin samples from 31 Japanese women (the mean body mass index [BMI] 23.9 ± 3.2 kg/m^2^, mean age 49.5 ± 4.8) undergoing breast reconstruction surgery were analyzed. Gene expression levels of elastin-regenerative and -degradative molecules were assessed by quantitative polymerase chain reaction in the epidermis, dermis, and subcutaneous fat. Dermal elastic fiber content was evaluated histologically using Elastica van Gieson staining. **Results:** No statistically significant correlations between the BMI and elastin-degrading gene expression (*NE*, *MMP2*, *MMP9*, and *NEP*) were observed. *ELN* expression in the dermis showed a significant positive correlation with the BMI (ρ = 0.517, *p* = 0.003), potentially reflecting a compensatory response. Histological analysis revealed a significant inverse correlation between dermal elastic fiber content and the BMI (r = −0.572, *p* = 0.001), independent of age or smoking history. **Conclusions:** Even mild obesity is associated with the early degradation of dermal elastic fibers despite limited transcriptional alterations. These findings underscore the need for early skin care interventions to mitigate obesity-related skin fragility, especially in populations with predominantly mild obesity.

## 1. Introduction

Elastic fibers are critical components of the dermal extracellular matrix. In the lower reticular dermis, they are organized into thick, horizontally oriented bundles. In contrast, the papillary dermis contains thinner elastic fibers, known as oxytalan fibers, running perpendicular to those in the reticular dermis, which integrate into fibrillin-rich microfibrils at the dermo-epidermal junction [1,2,3]. Although elastic fibers constitute only approximately 2–4% of the dry weight of the skin [4,5,6,7], they play a critical role in maintaining the skin’s recoil capacity and strength, complementing the tensile strength of collagen fibers.

Obesity is widely recognized as a major public health concern and is associated with a range of skin complications. Previous studies have shown physiologic skin changes [8,9,10], delayed wound healing [11], increased vulnerability to bedsores [12,13], and notable skin laxity following bariatric surgery [14,15,16,17,18,19]). However, the specific effect of obesity on the dermal elastic fibers remains poorly understood. In our previous study using a spontaneously obese mouse model, we observed that obesity resulted in decreased skin tensile strength [20] and a reduction in dermal elastic fibers resembling age-related changes [21]. These alterations were accompanied by changes in gene expression associated with elastic fiber homeostasis, including reduced fibrillin-1 (FBN1) and increased neprilysin (NEP) expression [21]. FBN1, a critical component of extracellular microfibrils, serves as a scaffold for elastic fiber assembly and is expressed throughout life [22,23]. NEP is widely expressed in a variety of cells, and elevated plasma NEP levels have been reported in individuals with overweight and obesity [24]. Similarly, plasma and mesenteric fat NEP levels were increased in high-fat diet-induced obese mice [24]. These findings suggest that obesity-associated reductions in dermal elastic fibers may arise from both impaired regeneration and enhanced degradation processes. Supporting this, a human study by Ezure et al. revealed a significant inverse relationship between the body mass index (BMI) and dermal elastic fiber abundance in the abdominal skin of Caucasian females (the BMI 20–42 kg/m^2^) [25]. Interestingly, their findings suggested that, in humans, elastic fiber reduction due to elevated matrix metalloproteinase-9 (MMP9) secretion from hypertrophic subcutaneous adipocytes, which directly contributes to elastic fiber degradation. This differences in degradation mechanisms may reflect variations in obesity severity.

Although obesity is a global issue, its clinical definition varies across populations. In Japan, individuals with a BMI ≥ 25 kg/m^2^ are classified as obese, whereas according to the World Health Organization criteria, individuals with a BMI in the range of 25–30 kg/m^2^ are considered overweight. According to the 2023 National Health and Nutrition Survey in Japan, approximately 82% of Japanese individuals with a BMI ≥ 25 kg/m^2^ fall into the range of 25–30 kg/m^2^ [26]. Therefore, in this study, we defined mild obesity as a BMI of 25–30 kg/m^2^. While our previous studies have shown obesity-related gene expression changes in the skin of Japanese individuals [27,28], the impact on skin elastic fibers remains unclear.

To address this gap, the aim of the present study was to determine whether the structural deterioration of dermal elastic fibers occurs even in the context of mild obesity, as observed in the Japanese population. Specifically, we sought to (1) evaluate whether the expression of genes previously implicated in elastic fiber regeneration and degradation in mouse models is altered in human skin and (2) assess whether elastic fiber content in the dermis is reduced. Understanding its impact on dermal elastic fiber integrity is crucial, particularly in populations where this BMI range is not classified as obese according to international criteria.

## 2. Materials and Methods

### 2.1. Study Patients and Skin Sample Collection

We analyzed abdominal skin samples from women who had undergone breast reconstruction surgery at a hospital in Yokohama, Japan. An analysis of healthy skin samples from the same site within the lateral abdominal region in each patient was possible as breast reconstruction was performed after confirming no risk of tumor recurrence or metastasis. Patients with diabetes mellitus, infectious diseases, and systemic skin diseases (such as psoriasis and atopic dermatitis) were excluded. Patient characteristics, including height, weight, age, medical history, a history of chemotherapy, and medication on admission, were obtained from electronic medical records. The BMI was used as an indicator of obesity.

### 2.2. Tissue Processing

Tissue samples were processed immediately after breast reconstruction surgery. Samples were prepared for both molecular and histological analyses as described in the following sections. For molecular analysis, subcutaneous fat was carefully removed from beneath the dermis. Additionally, subcutaneous adipose tissue located directly beneath the dermis was collected for analysis. The defatted skin samples were cut into small pieces (2–3 mm) on ice and incubated with 2 U/mL dispase II in phosphate-buffered saline (PBS) at 4 °C overnight. Following incubation, the epidermis and dermis were separated and stored at −85 °C until RNA extraction. Subdermal adipose tissue samples were immersed in RNAlater™ Stabilization Solution (Ambion, Austin, TX, USA), incubated at 4 °C overnight, and then stored at −80 °C until RNA extraction.

Total RNA was extracted using the RNeasy Lipid Tissue Kit (QIAGEN, Hilden, Germany) according to the manufacturer’s instructions. RNA concentration was measured using a microvolume spectrophotometer (Q500; TOMY, Tokyo, Japan). Complementary DNA was synthesized using the PrimeScript™ RT Reagent Kit (Takara Bio, Shiga, Japan) according to the manufacturer’s instructions.

### 2.3. Quantitative Polymerase Chain Reaction

Gene expression levels were quantified using a real-time polymerase chain reaction (PCR) system (7900HT; Applied Biosystems, Carlsbad, CA, USA). PCR was performed using THUNDERBIRD^®^ SYBR qPCR Mix (Toyobo, Osaka, Japan). The thermal cycling conditions were as follows: initial denaturation at 95 °C for 60 s, followed by 40 cycles at 95 °C for 15 s and 60 °C for 45 s. The relative gene expression was quantified using the relative standard curve method. The expression levels were normalized to beta-actin (*ACTB*), which was selected as the most appropriate internal control after examining multiple candidate reference genes. The primers used in this study are shown in Table 1.

### 2.4. Histological Analysis

For histological analysis, skin samples collected immediately after breast reconstruction were fixed in 10% formalin (Wako Pure Chemical Industries, Ltd., Osaka, Japan), embedded in paraffin, and sectioned at a thickness of 5 μm. After deparaffinization and dehydration, the sections were stained with Elastica van Gieson (EVG) for microscopic examination. Two slides were prepared for each sample and stained. Three regions of reticular dermis from each slide were photographed using a light microscope (BX50, OLYMPUS, Tokyo, Japan). Quantification was performed using ImageJ software version 1.49 v (https://imagej.net/ij/ (accessed on 22 July 2025)) based on previous studies [25,29]. To ensure consistency, analysis areas were selected as follows: 100–350 µm below the epidermal basement membrane, free of skin appendages, and trimmed to 500 × 500 pixels (approximately 180 µm × 180 µm). Thresholding and binarization were applied to extract the elastic fibers, and their areas were calculated as a percentage of the unit area. The average of six measurements per sample was used for the analysis.

### 2.5. Statistical Analysis

Data are presented as the mean ± standard deviation and percentage. The Shapiro–Wilk test was used to assess the normality of the data distribution. For correlation analysis examining associations with elastic fiber content, either Spearman’s rank correlation coefficient or Pearson’s product moment correlation coefficient was applied depending on the results of the normality test. Student’s *t*-test was used for comparisons between two groups. All statistical analyses were performed using SPSS version 24 (IBM Corp., Armonk, NY, USA). Statistical significance was defined as a two-tailed *p*-value < 0.05. For post hoc power analyses, G*Power 3.1.9.2 (Franz Faul, University of Kiel, Kiel, Germany) was used.

## 3. Results

### 3.1. Patient Characteristics

The characteristics of the study participants (*N* = 31) are shown in Table 2. The mean age was 49.5 ± 4.8 years, and the mean BMI was 23.9 ± 3.2 kg/m^2^. Nine participants were classified as obese (BMI ≥ 25 kg/m^2^), according to the Japanese obesity criteria. Additionally, eleven patients had a history of smoking.

### 3.2. Elastin-Related Gene Expression and Its Association with BMI

Based on our previous mouse studies that identified molecules involved in elastic fiber degradation, we aimed to evaluate whether similar changes occur in Japanese individuals with mild obesity. To maintain a focused scope, we selectively analyzed the expression of genes previously implicated in elastin dynamics in mouse models to assess their relevance in human skin.

#### 3.2.1. Expression of Degradative Genes

The skin comprises three layers: the epidermis, dermis, and subcutaneous tissue. Molecules involved in elastic fiber degradation are expressed in each layer. Therefore, we measured the expression separately. Table 3 shows the results of a correlation analysis between mRNA expression levels and this BMI.

In the dermis, neutrophil elastase (NE) exhibited a weak positive correlation with the BMI (Spearman’s *ρ* = 0.280, *p* = 0.127, power = 33.8%). In the subcutaneous tissue, MMP9 also showed a weak positive correlation (Spearman’s *ρ* = 0.300, *p* = 0.101, power = 38.3%). However, none of the degrading enzymes showed a statistically significant correlation with the BMI in any skin layers.

In the epidermis, although NEP localization was confirmed and increased expression had been observed in obese mice, NEP mRNA levels were below the detection limit in most human samples. Thus, a quantitative analysis was not feasible.

#### 3.2.2. Expression of Regenerative Genes

In the dermis, key molecules involved in elastic fiber synthesis were detected. Among these, ELN (the elastin gene) expression levels showed a significant positive correlation with the BMI (Spearman’s *ρ* = 0.517, *p* = 0.003, power = 86.9%; Table 4). FBN1 and lysyl oxidase (LOX) also demonstrated a weak positive correlation (Spearman’s *ρ* = 0.303, *p* = 0.098, power = 38.8%; Spearman’s *ρ* = 0.237, *p* = 0.199, power = 25.2%; Table 4) that was not statistically significant.

Although several elastin-related genes previous identified in mouse models were examined, no significant transcriptional alterations were observed in the skin of Japanese individuals. These findings raised the possibility that structural changes may occur independently of transcriptional alterations.

### 3.3. Early Structural Deterioration of Dermal Elastic Fibers with Mildly Increased BMI

**Figure 1:**
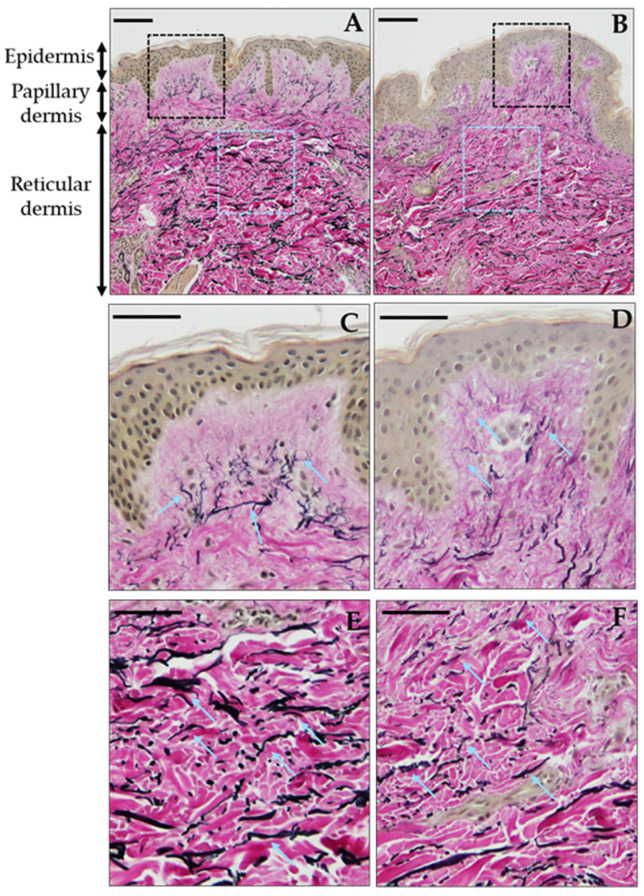
BMI-associated reduction in reticular dermal elastic fibers. Elastica van Gieson staining of skin sections (non-thresholded images). Elastic fibers appear black, collagen is stained red, and other tissue components are yellow. Cyan arrows indicate representative elastic fibers. Representative images are shown from individuals with lower ((**A**,**C**,**E**): 40 years old, BMI 20.5 kg/m^2^) and higher ((**B**,**D**,**F**): 41 years old, BMI 28.6 kg/m^2^) BMI values. (**A**,**B**) Low-magnification images (scale bar = 100 µm). Dashed boxes indicate magnified regions: black for papillary dermis (**C**,**D**) and cyan for reticular dermis (**E**,**F**). (**C**,**D**) Higher magnification of papillary dermis (scale bar = 50 µm). (**E**,**F**) Higher magnification of reticular dermis (scale bar = 50 µm).

Strikingly, despite the relatively narrow BMI range, distinct morphological changes in dermal elastic fibers were observed. This suggests that even mild increases in the BMI may induce the early structural deterioration of the skin’s elastic network. To further investigate this, elastic fiber morphology and quantity was evaluated using EVG staining. In the papillary dermis, fine elastic fibers were oriented vertically toward the epidermis. In contrast, in the reticular dermis, elastic fibers ran parallel to the skin surface. Notably, in the reticular dermis, elastic fibers became increasingly fragmented and showed a marked reduction with rising BMI values (Figure 1).

**Figure 2:**
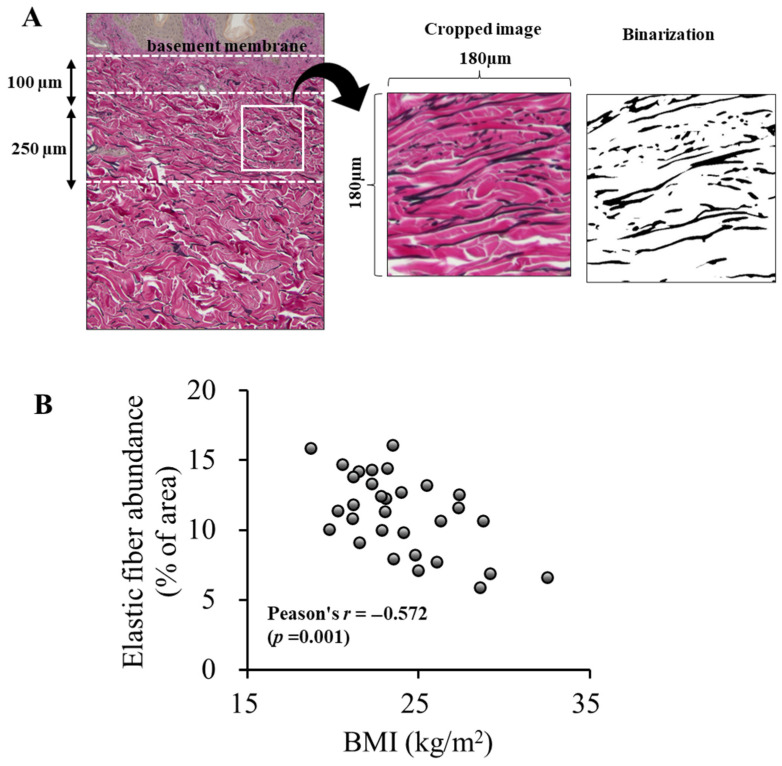
A quantitative analysis of dermal elastic fiber content and its association with the BMI. (**A**) A schematic illustration of the image analysis procedure. The images were cropped from a consistent area within the reticular dermis, maintaining a fixed size. The cropped images were then binarized for a quantitative assessment of the elastic fiber area. (**B**) The correlation between elastic fiber content in the reticular dermis and the BMI. A significant negative correlation was observed, indicating that a higher BMI is associated with reduced elastic fiber density in the reticular dermis. Abbreviation: BMI, body mass index.

To quantify elastic fiber content, a previously reported method was adopted [25,29] and the reticular dermis was selected as the region of interest. Elastic fiber content was quantified as the area of elastic fibers per unit area of dermis. This analysis revealed a moderate but statistically significant negative correlation between elastic fiber content and the BMI (Figure 2; Pearson’s *r* = −0.572, *p* = 0.001, power = 93.8%). No correlation was observed between elastic fiber content and age (Pearson’s *r* = 0.100, *p* = 0.593). Additionally, no significant difference in elastic fiber amount was observed between smokers and non-smokers (*p* = 0.228). Furthermore, among the participants, 21 individuals had received hormone therapy for breast cancer. Therefore, we compared the elastic fiber content between participants with and without hormone therapy to assess its potential influence on dermal structure and found no significant differences (*p* = 0.188).

These findings collectively suggest that structural alterations in dermal elastic fiber may occur prior to or independently of measurable gene expression changes, highlighting an early, potentially underrecognized impact of the BMI on skin architecture.

## 4. Discussion

### 4.1. Dermal Elastin Alterations in Mild Obesity

This study was conducted to investigate the early impact of mild obesity on elastic fiber homeostasis in the skin of Japanese individuals, with a particular focus on gene expression and histological changes. Our previous studies of obese mouse models have identified specific genes that are transcriptionally regulated in response to increased adiposity [21]. However, the current findings suggest that such clear transcriptional signatures may not be detectable in the context of mild obesity in humans. Nonetheless, we observed distinct structural alterations in dermal elastic fibers associated with the BMI, indicating that morphological deterioration may precede transcriptional changes.

### 4.2. Structural Degradation Despite Limited Transcriptional Changes

Despite conducting a layer-specific analysis, none of the examined elastin-degrading enzymes, including NE, MMP2, MMP9, and NEP, showed statistically significant correlations between their expression levels and BMI in any skin layer. Although weak positive correlations were observed for NE in the dermis and MMP9 in the subcutaneous tissue, neither reached statistical significance.

In our previous study of obese mice, we observed a significant reduction in elastic fibers within the papillary dermis [21]. This change was associated with a decreased expression of FBN1 and an increased expression of NEP. Furthermore, obesity induced by a high-fat diet resulted in elastic fiber reductions in both the papillary and reticular dermis [21]. FBN1 is a key scaffolding protein essential for elastic fiber assembly and is abundant in oxytalan fibers within the papillary dermis [2]. Therefore, increased NEP expression from both the epidermal and subcutaneous sides may have contributed to the earlier loss of elastic fibers in this region.

Unlike in the obese mouse model, human data showed more prominent degradation in the reticular dermis. This may be partly explained by the extremely low—often barely detectable—NEP expression in the epidermis. Additionally, NEP selectively degrades old or damaged elastin but not young, intact elastin [30]. Given this context-dependent elastolytic role, mild obesity may already damage elastic fibers to a point where they become more susceptible to degradation by baseline NEP activity. This may help explain why elastic fiber loss can occur in mild obesity despite the absence of significant increases in the expression of elastin-degrading enzymes.

In a study of Caucasian individuals with obesity, increased MMP9 expression in subcutaneous adipose tissue was associated with elastic fiber reduction within reticular dermis [25]. Notably, MMP9 has been directly demonstrated to degrade both elastic fibers and FBN1 in human skin under ex vivo conditions [31], supporting its potential pathogenic role. Since MMP9 is upregulated in hypertrophic adipocytes [25], such elevation may be limited in mild obesity. Our current study suggests that the transcriptional regulation of elastin-degrading enzymes may not be markedly affected by modest increases in the BMI and may only become evident at more advanced stages of obesity.

Additionally, hormonal factors, such as estrogen levels, are known to influence elastin metabolism. Previous studies have suggested that the topical administration of 17β-estradiol increases the expression of tropoelastin and fibrillin-1 in skin tissue, promoting elastic fiber synthesis [32]. Conversely, ovariectomy, a model of estrogen deficiency, has been shown to increase elastase activity and reduce skin elasticity, suggesting enhanced elastin degradation [33]. Notably, no significant correlation was observed between age and elastic fiber content, suggesting that factors other than age and estrogen status may contribute to elastin reduction.

Recent studies have indeed highlighted histological and inflammatory changes in the skin following bariatric surgery in individuals with severe obesity [34,35]. While surgical weight loss often improves obesity-related skin conditions such as striae, acanthosis nigricans, and acrochordons, skin elasticity frequently remains compromised [36], leading to sagging and the need for body contouring procedures. Our finding that mild obesity may already damage elastic fibers to a point where they become more susceptible to degradation by baseline NEP activity may partly explain why elastin loss and skin sagging persist even after significant weight reduction.

### 4.3. Compensatory Upregulation of ELN Concurrent with Elastic Fiber Loss

**Figure 3:**
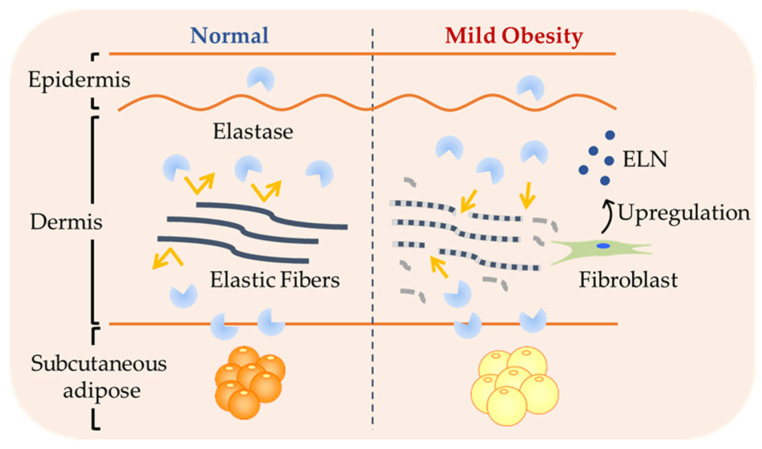
Schematic diagram summarizing the hypothesized mechanism of early elastic fiber degradation. Our results suggest that mild obesity may cause early damage to elastic fibers, making them more vulnerable to degradation by baseline elastase activity. Future studies should focus on elucidating the mechanisms underlying increased elastin vulnerability despite unchanged enzyme expression.

Among the elastin-regenerative genes examined in the dermis, only ELN expression showed a statistically significant positive correlation with the BMI. In human skin fibroblasts, elastic fiber synthesis is most active during the late fetal and early neonatal stages and declines significantly with age [37,38]. However, in response to injury, elastin gene expression can be reactivated [39,40]. Therefore, the observed upregulation of ELN in mild obesity may reflect a compensatory response to early tissue stress. Interestingly, our histological analysis revealed a clear and significant negative correlation between elastic fiber content and the BMI in the reticular dermis. These results point to a potential temporal dissociation between molecular responses and structural outcomes in the skin. That is, although molecular markers of degradation or regeneration may not yet show substantial changes, the physical integrity of the elastic fiber network is already compromised (Figure 3).

Moreover, the continued degradation of elastic fibers suggests that their loss may be progressing beyond the compensatory capacity of ELN upregulation. Due to the extremely low and limited turnover of elastic fibers, newly produced elastin is used for protection rather than replacing existing fibers [7,41,42]. These findings suggest that skin aging and fragility may commence even at the stage of mild obesity, highlighting the importance of early intervention.

Given the extremely slow turnover of elastic fibers, once degraded, they are unlikely to be restored even with lifestyle modifications, weight management, or skincare interventions. While agents such as estrogen derivatives are known to stimulate elastin synthesis, our data showed a compensatory upregulation of elastin expression. This indicates that increased elastin production alone may not be sufficient to prevent fiber loss. Therefore, we believe that protecting existing elastic fibers should take precedence over merely promoting their synthesis. Although specific preventive strategies cannot yet be proposed, our findings point to a current lack of understanding of early fiber degradation and outline key directions for future research. Future studies should focus on elucidating the mechanisms underlying increased elastin vulnerability despite unchanged enzyme expression, as this may provide key insights for prevention strategies. Furthermore, the findings underscore the need for clinical attention—even in mild obesity—particularly in regard to optimizing postoperative skin healing and managing potential skin-related complications.

### 4.4. Limitations

First, all participants were female patients undergoing breast reconstruction surgery. As a result, the cohort may not fully reflect the general population, and we cannot rule out the possibility of selection bias due to shared characteristics such as cancer history, treatment (e.g., chemotherapy or hormone therapy), or health-related behaviors. These factors might have influenced skin structure and gene expression profiles, potentially confounding the observed associations. Therefore, caution is warranted when generalizing the findings beyond this specific patient population.

Second, this study was limited to Japanese females, and racial differences in obesity-related tissue changes could influence the observed results. Future studies should include diverse populations to explore potential ethnic variations in the effects of mild obesity on dermal elastic fiber integrity.

Third, only mRNA expression was analyzed. The protein expression level and enzymatic activity were not evaluated. As protein levels and enzymatic activity do not necessarily correlate with mRNA levels, future studies should aim to assess the protein expression and functional activity of elastin-degrading enzymes to better understand their contribution to elastic fiber degradation.

Finally, the correlation between gene expression and the BMI was weak, suggesting that a larger cohort may be needed to detect more subtle transcriptional changes or biologically relevant effects. Although the sample size was sufficient to demonstrate significant alterations in elastic fiber content and ELN expression, especially for genes with weaker correlations, our results should be interpreted with caution. In addition, as a cross-sectional observational study, our design does not allow for causal inferences.

Despite these limitations, our study provides clear evidence that dermal elastic fiber reduction occurs even in individuals with mild obesity. These insights highlight the need for strategies to maintain skin quality in patients with obesity, particularly in the context of surgical interventions such as bariatric or oncologic procedures.

## 5. Conclusions

Although no robust BMI-associated transcriptional changes in elastin-related genes were detected, the observed reduction in dermal elastic fiber content in individuals with mild obesity underscores the sensitivity of the skin’s extracellular matrix to subtle metabolic alterations. These findings highlight the early and potentially underrecognized impact of mild obesity on skin structure. Our observations may have implications for the prevention of obesity-related skin aging and dysfunction, and they underscore the need for clinical attention.

## Figures and Tables

**Table 1:** Primer sequences for quantitative PCR analysis.

Target Gene	Forward Primer	Reverse Primer
*ACTB*	5′-ATTGCCGACAGGATGCAGA-3′	5′-GAGTACTTGCGCTCAGGAGGA-3′
*ELN*	5′-GAGTGAAGCCTGGGAAAGTG-3′	5′-ACTCCTGCTCCAGTGGGAAC-3′
*FBN1*	5′-CTTGAAGGGAGAAGGGCTGG-3′	5′-AGGGACCACTCGGACGCATA-3′
*LOX*	5′-ACTGCACACACACAGGAGTTG-3′	5′-GCCTTCTAATACGGTGGAAATT-3′
*LOXL1*	5′-GACTTCGGCAACCTCAAGC-3′	5′-TGTTGCAGAACAAGTCGGAC-3′
*NE*	5′-GGGCTACCCCGGATGC-3′	5′-GCGTTGGTAGTAGAGTCGATC-3′
*NEP*	5′-CCTTCTTTAGTGCCAGGCAG-3′	5′-TGAGTCCACCAGTCAACGAG-3′
*MMP2*	5′-CCTGAGATCTGAGCCAGGACAT-3′	5′-GCCAAATGGAACCGGTGCCTT-3′
*MMP9*	5′-TTCATCTTCACCCGAGACATC-3′	5′-CTTGTGCGTGTCAAAGTTCG-3′

**Table 2:** Patient characteristics (*N* = 31).

Characteristic	*N*	Average ± SD, *n* (%)
Age (year)	31	49.5 ± 4.8
BMI (kg/m^2^)	31	23.9 ± 3.2
Obese (BMI ≥ 25)	31	9 (29.0)
Smoking history	25 ^1^	11 (44.0) ^1^

**^1^ Unknown (*n* = 6). BMI, body mass index; SD, standard deviation.**

**Table 3:** Correlation between elastin degradative gene expression level and BMI (*N* = 31).

Gene	Dermis		Subcutaneous Tissue	
	Correlation Coefficient	*p*	Correlation Coefficient	*p*
*NE*	0.280 ^2^	0.127	0.176 ^2^	0.343
*NEP*	0.034 ^1^	0.855	0.042 ^2^	0.822
*MMP2*	0.203 ^2^	0.274	0.140 ^2^	0.451
*MMP9*	0.034 ^2^	0.855	0.300 ^2^	0.101

**^1^ Pearson’s *r*, ^2^ Spearman’s *ρ*.**

**Table 4:** Correlation between elastin-regenerative gene expression level and BMI (*N* = 31).

Gene	Dermis	
	Correlation Coefficient	*p*
*ELN*	0.517 ^2^	0.003
*FBN1*	0.303 ^2^	0.098
*LOX*	0.237 ^2^	0.199
*LOXL1*	0.049 ^1^	0.792

**^1^ Pearson’s *r*, ^2^ Spearman’s *ρ*.**

## Data Availability

The raw data supporting the conclusions of this article will be made available by the authors on request.

## Abbreviations

The following abbreviations are used in this manuscript:

PCR	Polymerase chain reaction
EVG	Elastica van Gieson
BMI	Body mass index
ELN	Elastin gene
NE	Neutrophil elastase
NEP	Neprilysin
FBN1	Fibrillin-1
MMP	Matrix metalloproteinase
ACTB	Beta-actin
